# The Impact of Social Determinants on Pancreatic Cancer Care in the United States

**DOI:** 10.3390/cancers17121898

**Published:** 2025-06-06

**Authors:** Shreeja N. Patel, Joseph R. Habib, Daniel Brock Hewitt, Michael D. Kluger, Katherine Morgan, Ammar A. Javed, Christopher L. Wolfgang, Greg D. Sacks

**Affiliations:** Department of Surgery, New York University Langone Health, New York, NY 10016, USAmichael.kluger@nyulangone.org (M.D.K.); katherine.morgan@nyulangone.org (K.M.);

**Keywords:** pancreatic cancer, social determinants

## Abstract

**Background/Objectives**: Pancreatic cancer treatment is complex and requires involvement of multidisciplinary care teams. Despite the aggressive nature of pancreatic cancer and its poor survival rate, outcomes are often determined by whether patients can establish care and receive guideline-concordant treatment. Social determinants of health impact access to care and provide an explanation for the variability seen in treatment and outcomes across patients of different backgrounds. This review will focus on the impact of these social determinants in the United States, raise awareness, and propose actionable changes that have potential to positively impact pancreatic cancer care. **Methods**: A comprehensive narrative review was conducted to assess the impact of social determinants of health, particularly socioeconomic status, insurance status, race, and educational level, on disparities in pancreatic cancer care. **Results**: Financial insecurity, lack of insurance, low educational attainment, and minority race are associated with delays in diagnosis of pancreatic cancer, lower likelihood of treatment at high-volume centers, reduced access to surgical resection and chemoradiation, and worse outcomes. Efforts to address disparities should target improving patient experiences and expanding access to treatment at the individual patient level and system-wide. Potential measures include utilizing patient navigators and community health workers, simplifying patient communication, and facilitating cancer care at high-volume multidisciplinary centers. **Conclusions**: Socioeconomic status, insurance status, race, and educational level influence the entire pancreatic cancer care continuum from diagnosis to outcomes after treatment.

## Simple Summary

Pancreatic cancer is associated with a dismal 5-year survival rate. Despite the advent of more effective multimodal chemotherapies and novel surgical techniques, outcomes remain poor. This is largely attributed to delay in diagnosis given only one in every five patients has resectable disease at the time of presentation. Social determinants of health, namely economic stability, education, race, and insurance status, also play an important role in cancer-related outcomes. Initiatives to address the disparities in pancreatic cancer care target one or more social determinants. These initiatives focus on expanding access to quality multidisciplinary care for all patients with pancreatic cancer regardless of cost, insurance status, education level, and race.

## 1. Introduction

With a rising annual incidence, pancreatic cancer is now the third leading cause of cancer-related mortality in American men and women [1]. Despite the advent of more effective multi-agent chemotherapies and innovative surgical strategies that have increased surgical candidacy, the 5-year survival rate remains poor at approximately 13% [2,3,4,5]. This poor survival rate can be explained by both the lack of available screening tests and the often-asymptomatic nature of pancreatic cancer resulting in advanced disease at diagnosis.

Despite generally poor outcomes in patients with pancreatic ductal adenocarcinoma (PDAC), significant disparities exist across patients from diverse backgrounds. Optimizing outcomes in PDAC requires complex, multidisciplinary care, making delays in treatment and barriers to accessing care particularly consequential. Several social determinants exacerbate these challenges and worsen the already dismal outcomes in patients with PDAC. The most impactful of the social determinants include socioeconomic status (SES), education, race, and insurance status [6]. These social elements are interconnected and perpetuate disparities because patients who face structural barriers will persistently be unable to access the resources needed to optimize their health, despite medical advancements. Social factors influence everything from health behaviors to healthcare utilization. While SES, education, race, and insurance status may be upstream from cancer care, they inevitably affect treatment and outcomes [7,8]. Inequalities can manifest as delays in diagnosis and treatment or the receipt of substandard treatment, which can ultimately affect outcomes. This narrative review aims to summarize and synthesize the current literature on the impact that social determinants have on pancreatic cancer care and further highlight tangible target areas for improvement that may lead to better outcomes in vulnerable patient populations.

## 2. Materials and Methods

A narrative review was conducted to synthesize the evidence on the impact of social determinants of health on pancreatic cancer care in the United States. The methodology was guided by principles of transparency and replicability, based on the PRISMA statement [9].

Search Strategy

Articles were identified via PubMed, Cochrane, and Embase on 9 February 2025, using the keywords “pancreatic cancer” and “social determinants of health” and MeSH terms “Carcinoma, Pancreatic Ductal” and “Socioeconomic Disparities in Health”. Studies related to non-pancreatic gastrointestinal cancers, performed outside the United States, or including qualitative data were excluded. To ensure comprehensiveness, references from relevant studies were reviewed for additional sources.

Inclusion and Exclusion Criteria

Studies were included if they examined the impact of one or more SDOH on any aspect of pancreatic cancer care. Exclusion criteria included the following: studies focused on non-pancreatic cancers; those conducted outside the United States; conference abstracts, editorials, or non-peer-reviewed publications; and non-English studies.

Study Selection

**Figure 1:**
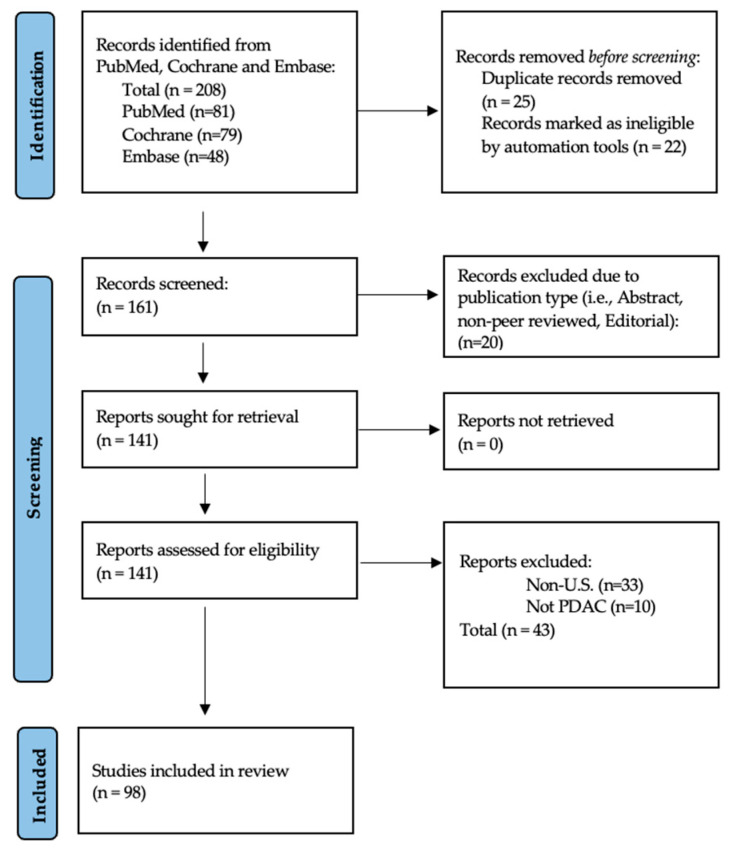
PRISMA flow diagram of selection process for reviewed studies.

The search yielded 208 records. After removal of duplicate studies, two reviewers (SNP and JRH) independently screened 10% titles and abstracts against the inclusion/exclusion criteria. One reviewer (SNP) screened the remaining studies following the rapid review methodology [10,11]. Any discrepancies were subsequently resolved through discussion. Full-text articles (n = 141) were retrieved and assessed for eligibility, resulting in 98 studies included in the final review. PRISMA flow diagram (Figure 1) illustrates the selection process.

Assessment of Study Quality

The validated Newcastle–Ottawa Scale (NOS) instrument for observational studies was employed to assess study quality. This tool evaluates scored studies based on selection (0–4), comparability (0–2), and ascertainment of exposure/outcome (0–3) [12].

Given that the reviewed studies not only utilized differing methodologies but also relied on different datasets, their findings are incommensurable. Therefore, when interpreting and combining findings of the reviewed papers, a qualitative approach was required in which two reviewers (SNP and JRH) considered a host of factors including sample size, validity, generalizability, relevance, and statistical significance of reported findings.

## 3. Results

Out of the studies reviewed (n = 98), the majority of studies included were observational (82%) and the remainder were review articles (12%) or prospective trials (6%). The average year of publication was 2020 and the median and mode year was 2022. Notably, the earliest year of publication for a reviewed study was 2005, with only five studies published prior to 2015. Using the NOS instrument to assess study quality, all observational studies were scored as moderate or good.

### 3.1. Socioeconomic Status

Socioeconomic status (SES), a measure of one’s combined social and economic status, is known to influence health outcomes [13]. With respect to cancer, financial stability not only determines what kinds of treatments patients have access to but also contributes to lived environments that make patients more or less vulnerable to developing cancer and experiencing progression. In pancreatic cancer, multiple studies have shown that socioeconomic factors, such as income, housing stability, employment, and food insecurity, can adversely impact delivery of treatment and thus outcomes [8,14,15,16].

For patients with resectable PDAC, financial insecurity is associated with lower odds of receiving neoadjuvant and multimodal therapy [14]. Patients with low SES with localized disease are less likely to undergo surgery and are more likely to be lost to follow-up after resection compared to patients of higher SES [15,16]. Furthermore, patients of low SES experience significant delays in treatment (OR 1.22, 95% CI [1.10–1.34], *p* < 0.05) and are ultimately less likely to receive adjuvant chemotherapy after resection (OR 1.30, 95% CI [1.20–1.41], *p* < 0.05) [17]. Given that surgical resection is the only chance for cure, and that adjuvant chemotherapy after resection prolongs survival, these patients often do not receive standard-of-care treatment [18]. Overall survival after surgery is significantly worse in patients of lower SES compared to patients of higher SES (median OS of 20.0 months for high SES vs. median OS of 17.0 months for low SES; *p* < 0.001) [8]. Furthermore, achievement of a textbook outcome (TO) after surgery (absence of postoperative surgical complications, prolonged length of stay, 90-day readmission, and 90-day mortality) is significantly less likely in patients of lower SES (OR of achieving TO in high SES = 0.94, 95% CI [0.87–1.0] vs. OR of achieving TO in low SES = 0.89, 95% CI [0.82–0.97]) [19].

SES is also a driving factor of the kind of treatment center that patients with PDAC can access. This is important, as treatment at high-volume centers (HVCs), defined as those performing at least 20 pancreatectomies per year, is associated with fewer perioperative complications, superior lymph node yield, higher rates of chemotherapy delivery, shorter lengths of stay, and longer overall survival [20,21]. Furthermore, patients treated at non-academic and low-volume centers (LVCs) experience treatment delays on the order of days. While delaying treatment initiation by days may seem inconsequential, ideally, pancreatic cancer treatment should be delivered expeditiously given delays may influence treatment options and thus patient prognosis [22]. When patients are initially diagnosed and treated at an LVC, subsequent referral to a HVC leads to improved outcomes (OS of LVC referred to HVC for locoregional disease = 16.6 months, 95% CI [15.3–17.9] vs. OS of LVC only for locoregional disease = 8.2 months, 95% CI [7.7–8.7], *p* < 0.001) [23]. However, patients of lower SES are ultimately even less likely to be referred to high-volume centers after initial presentation [23,24].

Rural patients of lower SES are at a particular disadvantage as they often live far from the nearest hospital [25]. Rural residence is thus an independent predictor of later stage of PDAC at diagnosis, which is unsurprisingly exacerbated by sparse primary care networks in these areas [26]. Furthermore, traveling for pancreatic cancer treatment is not inconsequential as traveling >12.5 miles for resection is associated with worse overall survival. Patients experience worse outcomes when traveling to receive care at academic centers that perform fewer than 20 pancreatectomies per year compared to traveling to receive care at centers that perform these operations more frequently [24,27]. Thus, patients of lower socioeconomic status residing in rural areas are particularly susceptible to experiencing shorter overall survival after resection of pancreatic cancer (5-year rural OS 18.8%, 95% CI [17.4–20.2%] vs. 5-year urban OS 22.3%, 95% CI [21.6–22.9%], *p* < 0.0001) [28].

Food insecurity is a known direct consequence of socioeconomic adversity as households with lower income are more likely to be food insecure [29]. An analysis of the impact of food insecurity on outcomes of patients with hepatobiliary cancers who underwent surgical resection found that patients who lived in counties with higher food insecurity were more likely to experience perioperative complications and be at higher risk of 90-day mortality (OR 0.69, 95% CI [0.54–0.88], *p* = 0.003) [30].

In culmination, median overall survival for PDAC is associated with ~6 month longer survival for patients of high SES compared to those of low SES for all stages of PDAC except metastatic disease, likely due to the universally poor outcomes observed when pancreatic cancer spreads to other organs (median OS low SES = 14.1 months, 95% CI [13.4–14.8] vs. median OS high SES 20.2 months, 95% CI [19.6–20.8]) [31,32].

### 3.2. Insurance Status

A lack of health insurance is a known barrier to receiving cancer care in the United States. The uninsured are disadvantaged at every stage of workup and management and are more likely to present with advanced cancer at diagnosis [33]. Insurance type also has important implications. For example, cancer patients with Medicare or Medicaid suffer worse outcomes and reduced survival compared to those who are privately insured (pancreatic cancer; Medicare and Medicaid insured vs. privately insured HR: Stage I = 2.86 95% CI [2.14–3.82], Stage 2 = 1.72, 95% CI [1.50–1.99], Stage 3 = 1.00, 95% CI [0.81–1.24], Stage 4 = 1.59, 95% CI [1.43–1.77]) [34].

In pancreatic cancer, health insurance status correlates with disease burden at diagnosis. Accordingly, uninsured patients are more often diagnosed with pancreatic cancer at later stages, rendering them unresectable [6,35,36]. Large database studies underscore this finding, demonstrating that private health insurance significantly decreases a patient’s likelihood of being diagnosed with stage IV pancreatic cancer (private vs. no insurance = OR 0.72, 95% CI [0.67–0.74]) [37]. Policies that have sought to improve access to health insurance, such as Medicaid expansion under the Affordable Care Act, have resulted in earlier stage at diagnosis for Medicaid or uninsured pancreatic cancer patients (stage I/II, difference in differences = 4.71, *p* = 0.03) [38]. Furthermore, patients in states with Medicaid expansion have superior operative outcomes and overall survival compared to those residing in states without Medicaid expansion (HR = 0.82; 95% CI 0.73–0.90; *p* < 0.001) [39].

Uninsured or publicly insured patients do not receive the same degree of treatment for pancreatic cancer compared to their insured counterparts. Private insurance is associated with more treatment, regardless of the treatment modality (OR = 1.41; *p* < 0.001) [40]. Alternatively, patients who lack health insurance or on Medicaid are significantly less likely to receive neoadjuvant (uninsured: OR = 0.55 95% CI [0.36–0.85], *p* < 0.01) and multimodal therapy (uninsured: OR = 0.58, 95% CI [0.42–0.79], *p* < 0.01; Medicaid: OR = 0.54, 95% CI [0.44–0.65] *p* < 0.001) [14,41]. This is particularly alarming considering access to these treatments may not only help control systemic disease but are also critical in increasing surgical candidacy in patients with locally advanced cancer [42]. For those with resectable PDAC, having private insurance is also a predictor of undergoing surgery [6] while uninsured patients undergo resection at significantly lower rates (OR = 0.07, 95% CI [0.01–0.49]) [43]. Further, when patients that are publicly insured undergo pancreatoduodenectomy for PDAC, they have a significantly longer length of stay (LOS) (8 versus 7 days, *p* = 0.021) and are more likely to experience postoperative complications compared to those that are privately insured [19,44]. Patients with Medicare (OR 1.660; 95% CI 1.123–2.454) or private insurance (OR 1.597; 95% CI 1.090–2.340) are more likely to undergo minimally invasive surgical resection [45]. Taken together, insurance coverage significantly increases the likelihood of undergoing surgical resection in multiple patient cohorts, a mandatory prerequisite for potential cure.

### 3.3. Educational Background

Higher educational attainment is associated with a healthier lifestyle and prolonged survival [46]. Not only does educational achievement create a pathway towards employment and financial security that allows individuals to access superior healthcare, but it also encourages engagement in healthier behaviors that may decrease susceptibility to a variety of diseases [47]. For example, cigarette smoking is a known modifiable risk factor for pancreatic cancer that is associated with increased incidence of PDAC and worse outcomes [48,49,50]. Individuals with lower levels of educational attainment are more likely to smoke cigarettes and be less successful in attempts to quit [51]. Though the underlying etiology of pancreatic cancer is multifactorial, the relationship between smoking and education level is pertinent, as efforts to expand access to education have the potential to reduce engagement in unhealthy behaviors that contribute to the development of this deadly cancer.

In PDAC, education level also impacts the timing of when patients receive treatment, what treatments are offered, and how patients navigate available therapeutic options. Completing higher education is associated with initiating treatment earlier than those with lower levels of educational attainment (for population quartile with the lowest percentage of patients without a high school degree compared to the quartile with the highest percentage: HR 1.09; *p* < 0.001) [40]. Furthermore, lower education level is associated with lower likelihood of receiving guideline-concordant care across all stages of pancreatic cancer (percentage of patients receiving guideline-concordant care in most educated vs. least educated quartile: 26.8% vs. 15.1%, *p* < 0.001) [52]. There is also an association between high school completion and the hospital setting in which patients seek surgical excision. Those that have graduated high school are more likely to be treated at academic medical centers when undergoing surgery, where perioperative outcomes are superior (percentage receiving surgery at academic centers in highest proportion of high school graduates vs. lowest proportion of high school graduates: 26.9% vs. 16.2%, *p* < 0.001) [40]. 

Health literacy parallels educational level. Achieving a higher educational level is associated with a greater ability to digest and act on health information [53]. Patients newly diagnosed with pancreatic cancer are typically presented with multimodal treatment options that are complex and often unfamiliar. Lower health literacy is associated with communication difficulties, as patients may struggle to understand written materials and conversations with their providers. This limits patient engagement and negatively impacts shared decision-making [54]. Patients with greater degrees of health literacy want to actively participate in surgical decision-making in premalignant lesions such as intraductal papillary mucinous neoplasms that can progress to pancreatic cancer [55]. Therefore, improving health literacy has the potential to empower pancreatic cancer patients to engage with their providers and actively participate in their cancer care. Cancer patients with more active roles are more satisfied with their care and can have better health outcomes [56].

Overall, level of educational attainment is an important mediator of how patients with pancreatic cancer establish care, receive treatment, and navigate recovery after undergoing surgical resection or receiving multimodal therapy.

### 3.4. Race

The incidence of PDAC is reported to be 50–90% higher in Black patients than other racial groups [57]. In terms of baseline characteristics, queries into the Surveillance, Epidemiology, and End Results (SEER) database have shown Black patients are generally younger than White patients at the time of diagnosis [58,59]. Observational data has also shown that in Black patients, stage of PDAC at diagnosis is also more advanced [60]. Poulson and colleagues looked more granularly at race distribution across neighborhoods and disparities in pancreatic cancer. Their analysis of the SEER database found that Black patients living in more segregated neighborhoods are more likely to present at an advanced stage than White patients living in the least segregated neighborhoods (RR = 1.12, 95% CI [1.08–1.15]). Notably, their study did not control for other social determinants of health, such as income, insurance status, educational achievement, and employment status [36].

Despite being diagnosed at a later stage, a National Cancer Database analysis that adjusts for SES suggests that Black patients also tend to obtain less overall treatment (OR = 0.97, *p* = 0.04) and experience more treatment delays (HR = 0.89, *p* < 0.001) [40]. Across all stages of pancreatic cancer, when adjusting for other social determinants, it has been observed that amongst patients who did not receive standard-of-care treatment, a significantly higher percentage were Black compared to patients who did receive standard-of-care treatment (12.6% vs. 11.4%, *p* < 0.001) [52]. This disparity is observed regardless of the type of treatment, as Black patients are receiving less chemotherapy, radiation, and surgery for pancreatic cancer [40]. Given that radical surgical resection is the only curative treatment currently available, it is worth noting that even when Black patients are diagnosed with localized disease amenable to resection, they are less likely to undergo surgery (RR = 0.89, 95% CI [0.83–0.94]) [36]. An SEER study provides a potential explanation for these disparities as, when compared to White patients, Black patients were less likely to establish care with medical oncologists (AA 56.2% vs. white 60.2%, *p* < 0.001), radiation oncologists (AA 25.6% vs. white 32.5%, *p* < 0.05), and surgeons (AA 72.1% vs. white 78%, *p* = 0.01) [6]. An analysis of patients with resected PDAC in New York state from 2007–2017 also found that Black patients were more likely to undergo surgery at low-volume centers (OR 2.21, 95% CI: [1.69–2.88]) [22]. Interestingly, this study also found Black (50.4%) and White (52.6%) patients received significantly less surveillance imaging after resection compared to patients of Asian (73.9%) or Hispanic (61.9%) race/ethnicity (*p* = 0.01) [22]. When adjusting for other social determinants, surgeons appear to be less likely to recommend resection for Black patients (OR = 0.88, 95% CI: [0.82–0.95]) compared to comparably staged cancers in White patients (OR = 0.83, 95% CI: [0.76–0.91]) [61]. Further, as minimally invasive surgical (MIS) techniques become increasingly utilized by pancreatic surgeons, not all patients with pancreatic cancer are undergoing MIS resections equally. Black patients are more likely than White patients to undergo open surgical resections rather than robotic or laparoscopic resections (OR = 0.82; 95% CI: [0.70–0.96]) [45].

Of note, Black patients are significantly more likely to decline treatments offered to them for pancreatic cancer whether it is chemotherapy, radiation, or surgery. Refusal of treatment amongst minority patients is seen across other malignancies and is attributable to multiple factors including mistrust of the medical establishment and suboptimal provider communication [62,63,64]. With respect to exposure to novel treatment approaches via clinical trial participation, Black patients are more likely to be ineligible to participate when compared to White patients, and their exclusion is typically related to comorbidities such as renal or cardiac dysfunction and HIV, Hep B/C positive status rather than prior cancer treatment such as receipt of neoadjuvant chemotherapy (percentage of Black patients ineligible 42.4% vs. percentage of White patients ineligible 33.2%, *p* = 0.023) [65]. Even as overall enrollment in pancreatic cancer clinical trials increases, Black patients remain underrepresented [66].

Many of the disparities observed in Black patients with pancreatic cancer can also be seen in Hispanic patients. A retrospective analysis of patients with pancreatic cancer treated at a National Cancer Institute (NCI)-designated comprehensive center in Arizona found that Hispanic patients with early-stage PDAC present at a younger age compared to non-Hispanic patients (median age at diagnosis for Hispanic patients 60.7 yrs vs. 66.7 yrs in non-Hispanic patients, *p* = 0.03) [67]. Another study looking at data from the Cancer Surveillance Program of Orange County (California) and the San Diego Imperial Organization for Cancer Control found that, when compared to all other races, Hispanic patients were the most likely to receive no treatment regardless of stage (Hispanic = 56.9%, non-Hispanic white = 52.1%, African American = 52.0%, Asian = 48.6%) [60]. Further, Hispanic patients undergoing surgical resection are less likely to travel to receive treatment at academic programs (APs) where there are lower rates of perioperative complications (69% had surgery at an AP versus 76% of non-Hispanic patients, *p* < 0.001) [68]. Notably, patients of Hispanic origin are least likely to undergo MIS (Hispanic vs. non-Hispanic, OR = 0.24, 95% CI [0.07–0.79], *p* = 0.019) [6].

Disparities in pancreatic cancer outcomes also exist amongst Asian American patients. Some of these differences may be attributed to cultural differences across this heterogeneous ethnic group. Forms of communication, including different dialects, healthcare-seeking behaviors, and strength of community bonds vary across the Asian diaspora [69,70]. These aspects have the potential to shape whether patients seek out and comply with treatment after diagnosis. Some Asian American patients also prescribe to alternative or “Eastern” medicine practices. In cancer care, patients may only wish to receive alternative therapies and forgo conventional multimodal treatment regimens, which put patients at higher risk of poor outcomes [71,72,73]. Multivariate analyses demonstrate that Southeast Asians with pancreatic cancer are less likely to receive multimodal therapy and undergo surgical resection relative to all other Asian subgroups (chemotherapy: 28.7% vs. 32.1%, *p* < 0.001, radiation: 12.7% vs. 14.1%, *p* < 0.001, surgery: 21.4% vs. 23.8%, *p* < 0.001) [74].

The data remains sparse regarding outcomes and disparities in treatment for Native American patients diagnosed with pancreatic cancer. However, when American Indian or Alaskan Native patients reside in urban areas, their incidence rates of pancreatic cancer are significantly higher than those for non-Hispanic white patients living in the same areas (Native American: 26% vs. non-Hispanic white: 18%) [75].

Patients of all racial backgrounds can experience language barriers when navigating healthcare in the United States. Roughly 26 million Americans have limited English proficiency (LEP), meaning English is not their primary language and they have a limited ability to speak, read, write, or understand English [76]. Amongst those with LEP, the majority are Hispanic (62%) and over a fifth are Asian (22%) [77]. When patients have limited English proficiency (LEP) regardless of their racial background, their pancreatic cancer care may be impacted. A single-center retrospective study including 739 patients found that pancreatic cancer patients with LEP had significantly higher odds of death (HR 1.60, 95% CI 1.03–2.47) in a matched analysis. Further, patients with LEP were more likely to present with advanced-stage cancer and had lower odds of receiving guideline-concordant treatment [78]. Another single-institution retrospective study analyzing registry data, albeit with a smaller cohort (n = 155), found that in patients with advanced pancreatic cancer, LEP was not significantly associated with poorer overall survival (HR 1.42, 95% CI: 0.93–2.16, *p* = 0.103) [79]. The results of these studies suggest that, while efforts should be taken to adequately accommodate patients with LEP, further work is needed to sufficiently elucidate the relationship between English language proficiency and pancreatic cancer outcomes.

Overall, the literature reviewed suggests that race is a pertinent factor in the treatment patients with pancreatic cancer receive and significantly affects patient outcomes. An exception to this may exist amongst patients with metastatic PDAC. A single-center study found that time to treatment after diagnosis was not significantly associated with racial disparities or differences in outcomes. However, this study included a majority White (82.9%) and high SES study population, which likely biased its conclusions [80]. To that end, while many of the aforementioned studies control for other social determinants, particularly income, insurance status, and education level, a key limitation of the literature in this area is the inconsistent examination of these other factors in addition to race. Thus, the studies that exclusively look at race with respect to PDAC treatment and outcomes risk overstating the role of race by ignoring confounding social determinants and limit the generalizability of their conclusions.

## 4. Discussion and Future Directions

### 4.1. Existing Theoretical Framework and Areas for Future Study

Disparities in both care and outcomes exist for patients with PDAC across social determinants of health, including SES, degree of educational attainment, race, and insurance status in the United States. With the exception of race, these social determinants represent potentially modifiable risk factors with opportunities for improvement that can positively impact pancreatic cancer care. Given that our sourced articles originate from the United States, our article may not be generalized to other countries where access to healthcare and social dynamics differ.

The social determinants explored in our paper are not the only factors relevant in cancer care. Notably, gender differences, social support, caregiver involvement, community engagement, and immigration status are important determinants in cancer care worth mentioning. However, the literature significantly lacks a thorough discussion of how these factors inform pancreatic cancer care and outcomes. Gender differences have been shown to be significant in international studies looking at treatment allocation, survival by cancer stage, and outcomes in those with metastatic disease who received chemotherapy [81,82,83]. At present, there are no relevant domestic studies regarding gender differences in treatment allocation or outcomes in pancreatic cancer. In the case of social support, including both caregiver involvement and community engagement, the literature focuses primarily on caregiver burden and quality of life rather than how patients fare depending on their degree of social support [84,85,86,87,88]. Immigration status has been explored with respect to time to treatment in PDAC and was found to not be significantly associated with longer time to treatment after diagnosis [89]. Future studies should seek to further explore these other patient factors with respect to pancreatic cancer.

While we summarize the evidence on the impact of individual social determinants of health on various outcomes associated with pancreatic cancer, in reality, complex relationships between these determinants and the cancer care continuum contribute to observed disparities. A framework to understand these relationships developed by Alcaraz et al. considers how social and structural factors that are rooted in historic exclusion and discrimination inevitably produce environments that perpetuate inequality [7]. This can manifest as concentrated poverty, residential segregation, sparse public transportation systems, food deserts, failing school systems, and disproportionate exposure to pollution [90]. These environments then go on to not only reinforce structural and social injustice but also inform how patients ultimately navigate their cancer care [7]. Through healthcare delivery and quality, disparities in outcomes are ultimately observed as downstream consequences of social determinants of health.

The reviewed literature on social determinants in pancreatic cancer, particularly database studies, references individual factors such as educational level as proxies for social environment and social determinants. However, in doing so, the literature fails to account for the nuances that can exist between two individuals with the same degree of educational attainment. It is for this reason that an intersectional approach provides a wholistic framework through which pancreatic cancer care can be tailored to underserved populations [91]. Developed by critical race theorist Professor Kimberlé Crenshaw, intersectionality acknowledges that social determinants of health are not mutually exclusive and instead interact in complex ways [91,92]. For patients with pancreatic cancer, considering identities such as religion, immigration status, and language proficiency during every phase of cancer care has the potential to improve health outcomes for underserved patients.

In recognizing the advantages of an intersectional approach to understanding how interlocking patient identities inform disparities, questions arise regarding how this methodology fits with modern quantitative research practices. Furthermore, as we begin to better understand how intersectional identities influence cancer care and thus outcomes, we must then ask ourselves how we can best evaluate initiatives meant to address disparate outcomes.

### 4.2. Potential Interventions to Address Disparities

The following potential interventions to address disparities in cancer care include many that have only been shown to be effective in the context of malignancies other than pancreatic cancer. For these interventions, the literature lacks robust evidence for their effectiveness in reducing inequalities specifically in pancreatic cancer care. Thus, by presenting them here, we hope to encourage future work to look more closely at how their implementation can impact outcomes in pancreatic cancer.

Treatment at multidisciplinary centers (MDCs) may have the power to mitigate the disparities faced by patients from disadvantaged backgrounds. A study by Hoehn and colleagues showed that treatment at an MDC can improve access to neoadjuvant chemotherapy, MIS, and outcomes after surgical resection. Accordingly, low SES patients treated at an MDC experienced improved survival [93]. Similarly, minority patients have better outcomes when treated at integrated health facilities such as academic centers [39]. An instructive example is the safety-net hospital, which provides patients with a means to receive multidisciplinary and integrated healthcare regardless of their ability to afford care. Sridhar et al. report that patients with PDAC treated at an urban safety-net hospital with a focus on vulnerable patient populations were able to achieve comparable outcomes to national averages [89]. Thus, to address disparities amongst patients with pancreatic cancer, a concerted effort to support existing safety-net hospitals and open more of these centers in lower socioeconomic areas may be beneficial. Making this long-term initiative a reality would require buy-in from local and federal government as well as existing healthcare facilities to ensure that patients are referred to multidisciplinary centers after diagnosis.

Telemedicine allows patients to access care from the convenience of their homes. While treatment for PDAC necessitates either hospitalization or in-person visits, there is a role for virtual health in the post-treatment phase of the cancer continuum. Virtual visits can guide patients with exercise rehabilitation and nutritional optimization in the post-acute care setting. Telehealth programs designed for PDAC patients with a focus on rehabilitation and nutrition have demonstrated significant improvements in terms of quality of life and physical function regardless of cancer stage [94]. These programs may be of particular utility for patients residing in rural areas that must travel far distances to receive multidisciplinary care at academic centers. Healthcare providers are crucial stakeholders in expanding access to telemedicine. Their adoption of telemedicine in the short term can create long-term infrastructure within hospital systems for patients to access essential cancer care efficiently and conveniently.

The addition of patient navigators and community health workers to the care team is a means of addressing intersectional barriers in PDAC care. Patient navigation programs are community-based interventions aimed at providing individualized guidance for patients navigating their cancer care [95]. They are particularly effective at enhancing rates of initiation of and adherence to treatment in underserved patients with cancer [96]. When patient navigation is culturally tailored and educationally oriented, it not only improves adherence but also simultaneously improves a patient’s understanding of their diagnosis [97]. For racial and ethnic minority groups that have experienced discrimination, medical mistrust is prevalent and contributes to undertreatment and undesirable outcomes [98]. Community health workers (CHWs) are entrusted individuals already integrated into the community that have been shown to improve adherence to screening for breast, cervical, and intestinal cancers amongst patients of ethnic minorities [99]. A randomized control trial involving church-based CHWs showed Black patients who worked with CHWs were more adherent to screening protocols for colorectal cancer, suggesting that CHWs can successfully act as liaisons between patients and providers [100]. A similar model utilizing CHWs can be implemented for minority patients with PDAC to help engage these patients that may be hesitant to receive care. Creating programs that utilize both patient navigators and community health workers requires long-term investment from community institutions (i.e., churches, small businesses, etc.), healthcare systems, and providers who must work in tandem to ensure that patients are supported throughout their cancer care.

Given that education and health literacy are known determinants of outcomes for patients with PDAC, educational initiatives and efforts to simplify communication are worthwhile pursuits. For example, visual aids such as videos and animations can reach wide audiences and engage patients who may be alienated by purely written information. A retrospective study surveying viewers of Animated Pancreas Patient (APP) online modules found that participants reported significant knowledge gains and changes in their attitudes towards treatment after viewing the content [101]. In patients for whom English is not their primary language, simplified communications in the patient’s native language have been shown to bridge disparities in other solid organ cancers and should thus be considered in PDAC [102]. Culturally sensitive smartphone applications have also been successful in providing patients with accessible at-home educational content and facilitating communication with their healthcare teams [98,99]. A small trial including six patients with PDAC tested the use of a mobile application for daily symptom assessment and access to self-care advice in the first month after post-surgical resection discharge. The application also included real-time access to a nurse if alarming symptoms were experienced. Participants demonstrated ~80% adherence to daily symptom recording, suggesting similar apps are feasible for patients to use after discharge and enable patients to actively partake in their care when implemented [103]. However, like health literacy, digital literacy is an important consideration when utilizing smartphone-based interventions. Regardless of the format of educational initiatives or simplified communications, buy-in from healthcare providers is required to ensure that patients are made aware of these resources when navigating cancer care.

Expanding access to health insurance also has the potential to improve equitable access to pancreatic cancer care. For example, a study looking at rates of resection before and after Massachusetts health insurance expansion in 2006 found that compared to control states where there was no change, Massachusetts had increased resection rates [104]. Improvements in disparities seen in states with Medicaid expansion compared to those without expansion is additional evidence that increasing insurance coverage is a powerful tool for improving the equity of pancreatic cancer care [38]. Improving access to health insurance requires investment from not only the state and federal government but also private insurance companies that have the power to make insurance more affordable and accessible for individuals that otherwise experience barriers to care.

As the landscape of pancreatic cancer research continues to change and increasingly novel treatment strategies become available, it is important that all patients benefit from this innovation. Many of the aforementioned strategies, such as employing patient navigators and community health workers, simplifying trial communications, and expanding Medicaid coverage of trial costs, have the potential to increase enrollment amongst patients of racial and ethnically minority backgrounds [66].

## 5. Conclusions

In conclusion, pancreatic cancer care is impacted by social determinants of health including SES, insurance status, and educational attainment. Race also plays an important role; however, its effect in isolation is unclear given these social determinants are interconnected and influence every aspect of the cancer care continuum. Multiple possible interventions exist to address disparities and improve access to care for patients with PDAC (Table 1). To address disparities driven by SES, increasing access to safety-net hospitals would allow patients to access high-quality cancer care regardless of their financial situation. Furthermore, strengthening referral networks to tertiary and quaternary safety-net hospitals, including those with academic affiliations, can expose underserved patients to a wider range of services. Incorporating telemedicine may also provide an alternative for patients who cannot afford to travel for frequent appointments. Medicaid expansion would help bridge the gaps in care experienced by uninsured patients. For patients with limited educational attainment, utilizing visual aids and smartphone apps to facilitate and simplify communication has been shown to improve health literacy. Additionally, employing patient navigators has the potential to individualize cancer care so patients can be guided based on their particular needs. These patient navigators and community health workers can even act as cultural liaisons to aid in the delivery of culturally competent care, thereby addressing difficulties faced by patients of different racial backgrounds. Finally, targeted interventions to recruit diverse study populations for clinical trials can improve access to novel treatments in pancreatic cancer for historically underrepresented minorities.

## Figures and Tables

**Table 1:** Summary of social determinant related disparities and recommendations.

Social Determinants	Disparities	Possible Interventions
SES Race Education Level Insurance Status	Delayed diagnosis Delayed treatment Lack of treatment (surgery, chemotherapy, radiation) Longer LOS Perioperative complications Decreased survival	Treatment at MDC Safety-net hospitals Telemedicine Patient navigators Community health workers Culturally competent care Simplified communication Expanding insurance access Broadening clinical trial eligibility

## Data Availability

No new data was generated.

## Abbreviations

The following abbreviations are used in this manuscript:

PDAC	Pancreatic ductal adenocarcinoma
SES	Socioeconomic status
MDC	Multidisciplinary center
CHW	Community health worker
LOS	Length of stay
